# Nitro-oxidative nucleotide modifications in plants and associated microorganisms: signalling sensors or stress symptoms?

**DOI:** 10.1093/jxb/eraf188

**Published:** 2025-05-04

**Authors:** Jagna Chmielowska-Bąk, Ewa Sobieszczuk-Nowicka, Magdalena Arasimowicz-Jelonek

**Affiliations:** Department of Plant Ecophysiology, Faculty of Biology, Adam Mickiewicz University, Poznań, Uniwersytetu Poznańskiego 6, 61-614 Poznan, Poland; Department of Plant Physiology, Faculty of Biology, Adam Mickiewicz University, Poznań, Uniwersytetu Poznańskiego 6, 61-614 Poznan, Poland; Department of Plant Ecophysiology, Faculty of Biology, Adam Mickiewicz University, Poznań, Uniwersytetu Poznańskiego 6, 61-614 Poznan, Poland; Institut Sophia Agrobiotech, INRAE, France

**Keywords:** 8-Hydroxyguanosine, nitration/oxidation reactions, 8-nitroguanosine, reactive nitrogen species, reactive oxygen species, redox signalling

## Abstract

Reactive oxygen and nitrogen species (ROS and RNS, respectively) play crucial roles in the functioning of plants and associated microorganisms. These molecules are engaged in signalling and gene regulatory events, and affect, among others, developmental processes and multilevel responses to unfavourable conditions. The ROS/RNS effects are frequently dependent on the oxidation/nitration of biomolecules. The increasing number of reports provide evidence for the formation of nitro-oxidative modifications in nucleotides, although their exact roles in plants and microorganisms are still vague. It is still unclear if nitration/oxidation of nucleotides is a symptom of damage resulting from an altered nitro-oxidative status or a sensing/signalling element for metabolism adjustment. The present review discusses the consequences and possible biological functions of nitrated/oxidized nucleic acids and cyclic nucleotides in plants and microorganisms.

## Teaser

Nitro-oxidative nucleotide modifications are not only a symptom of damage but may also function as a sensor of the complex redox signalling network towards adjustment of metabolism in both plants and associated microorganisms.

## Introduction

The plant responses to stimuli, including interactions with beneficial or pathogenic microorganisms, depend on the activation of signalling pathways and changes in gene expression. In turn, gene expression can be modified by chemical, covalent modifications of nucleic acids. Methylation of cytosine in DNA (5mC) is a major epigenetic marker. The presence of 5mC affects chromatin structure and, in consequence, accessibility to the proteins involved in the process of transcription. This modification can have varying biological effects. In general, it is considered that 5mC present in the promoter regions inhibits gene expression. However, its formation in coding regions can lead to either stimulated or hampered transcription. Importantly, the pattern of DNA methylation can be passed to the offspring, leading to inheritable changes (reviewed in Czajka *et al.*, 2021; Kumar and Mohapatra, 2021). In the case of RNA, the most studied modifications include methylation of adenosine (m6A) and cytosine (5mC). The level of m6A is controlled by methylation and demethylation proteins, referred to as writers and erasers, respectively. In addition, this modification is recognized by the so-called readers, which mediate the downstream effects. In plants, m6A is present in 70–100% of transcripts, depending on the species and organelle. This modification is an important regulator of developmental processes, as highlighted by the fact that Arabidopsis mutants with decreased m6A levels show the embryo-lethal phenotype. In turn, 5mC is involved in regulating translation efficiency and translocating corresponding transcripts. Studies on Arabidopsis and rice revealed its pivotal role in tolerance to heat and oxidative stresses (reviewed in Chmielowska-Bąk *et al.*, 2019; Zhang *et al.*, 2020; Xiang *et al.*, 2024).

**Fig. 1.**
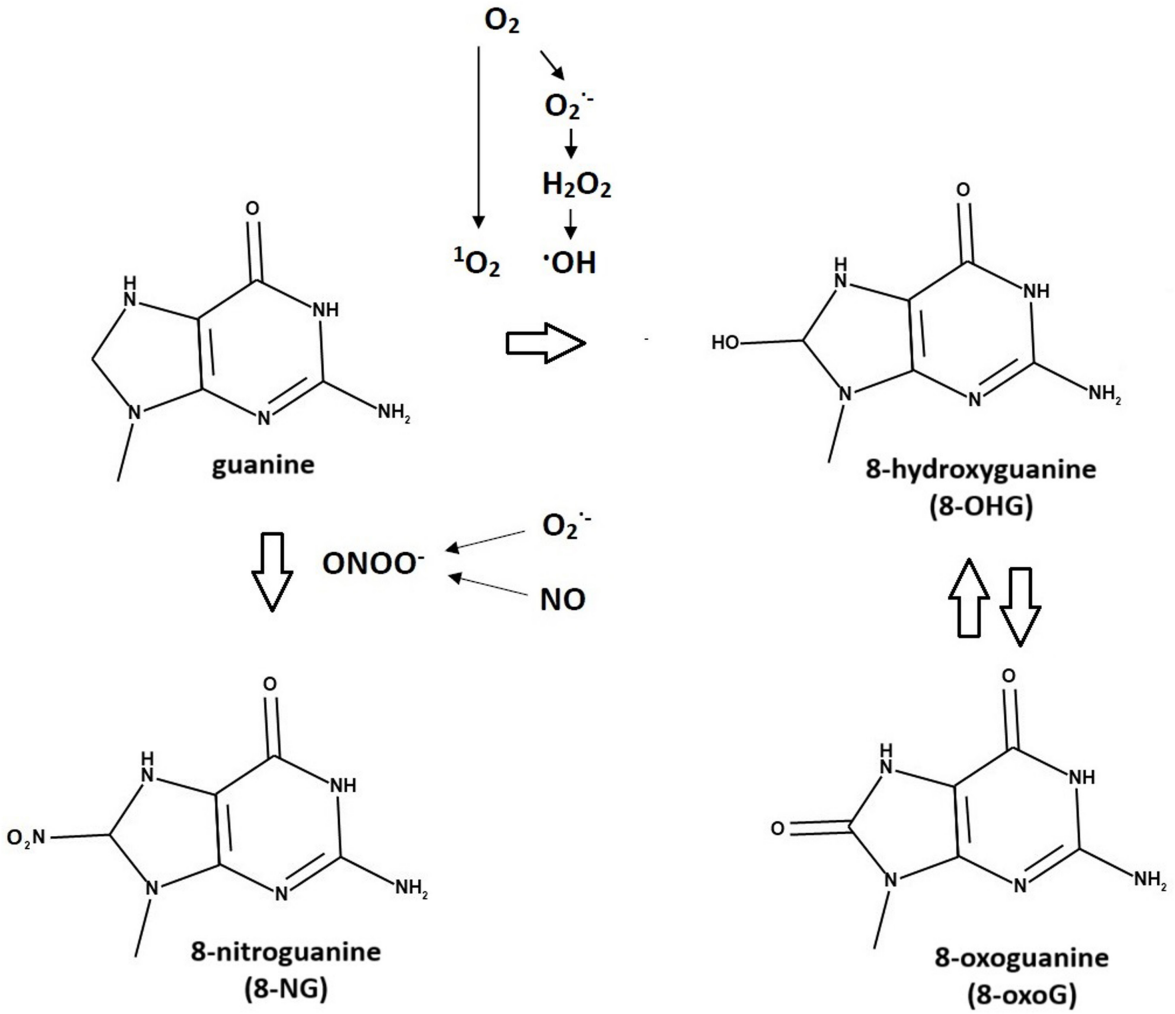
Chemical structure of the most frequent nitro-oxidative modifications. ROS, namely hydroxyl radical or singlet oxygen, mediate the formation of 8-hydroxyguanine (8-OHG) and its tautomer 8-oxoguanine (8-oxoG). The nitrating agent—peroxynitrite—mediates the formation of 8-nitroguanine (8-NG).

In addition to the enzymatically controlled methylation, nucleotides can be chemically modified by non-enzymatic reactions mediated by reactive nitrogen and reactive oxygen species (RNS and ROS, respectively). However, 5mC can be oxidized into 5-hydroxymethylcytosine (5hmC), 5-formylcytosine (5fC), and 5-carboxycytosine (5caC) sequentially by dioxygenases belonging to the ten–eleven translocation (TET) oxygenase family (Cadet and Wagner, 2014). RNS and ROS are commonly formed by‐products of an oxygen‐ and nitrogen‐rich environment. In optimal concentrations, these molecules exert important functions, such as acting as signalling molecules regulating a range of physiological processes (e.g. Considine *et al.*, 2015; Fichman *et al.*, 2023; Thiruvengadam *et al.*, 2024). Unfavourable conditions can lead to a disturbance in RNS/ROS homeostasis and enhanced formation of nitrated and oxidized derivatives. The RNS/ROS-mediated modifications of proteins and lipids are relatively well studied and mainly recognized as markers of nitro-oxidative stress (reviewed in Rubbo and Radi, 2008; Arasimowicz-Jelonek and Floryszak-Wieczorek, 2019; Akter *et al*., 2021; Corpas *et al*., 2021; Di Fino *et al.*, 2021). In contrast, relatively little is known about the RNS/ROS-modified nucleotides. However, due to its low one-electron redox potential, guanine (G) was recognized as the preferred target for oxidation and/or nitration reactions among purines (Thapa and Schlegel, 2015), leading to the formation of 8-hydroxyguanosine/8-oxoguanosine (8-OHG/8-oxoG) or 8-nitroguanosine (8-NG), presented in Fig. 1. Moreover, oxidized forms of 5mC, including 5hmC, 5fC, and 5caC, were detected within various phylogenetic groups of eukaryotes, such as, for example, ectomycorrhizal bicoloured deceiver *Laccaria bicolor* (Maire) P.D. Orton and saprotrophic gray shag [*Coprinopsis cinerea* (Shaeff.)], while the importance of individual oxidative cytosine modifications in epigenetic regulation was revealed (Ličytė *et al.*, 2022). The present review aims to present a discussion on recent findings on the occurrence and potential roles of nitro-oxidative modification of nucleic acids and cyclic nucleotides in plants and associated microorganisms, considering that nucleotide modifications may function as a specific chemical sensor for redox signalling during the contact between the host and the microorganism.

## Nitrative modifications of nucleic acids

The free radical nitric oxide (·NO) has been regarded as an important signalling molecule in living cells. Cellular oxidation–reduction reactions are the source of ·NO conversion to other redox forms that give rise to a further series of compounds, collectively known as RNS. Nitric oxide undergoes autooxidation reactions in the presence of O_2_, leading to the formation of nitrogen dioxide (·NO_2_), an oxidizing and nitrating agent; however, under normal conditions this process is rather slow (Groß *et al*., 2013). The radical ·NO_2_ can also arise from the nitrite that can be oxidized by the haem peroxidases in the presence of hydrogen peroxide (H_2_O_2_) or from the decomposition of peroxynitrite (ONOO^–^) (van der Vliet *et al*., 1997; Pryor *et al*., 2006; Groß *et al*., 2013). The latter RNS, ONOO^–^, is formed in one of the most rapid reactions known in biology between ·NO and the superoxide, and it was shown to be a strong oxidant and nitrating species (Prolo *et al*., 2024). Although within the cellular environment ONOO^–^ is a relatively short-lived molecule, it may readily migrate through biological membranes and interact with biomolecules in the surrounding cells within the radius of one or two cells (~5–20 µm) (Szabó *et al.*, 2007; Möller and Denicola, 2024). An increasing number of studies have reported that the nitrating agent cannot be considered as only a mediator of cellular dysfunction, but it also behaves as a potent modulator of the redox regulation in various cell signal transduction pathways, mainly through post-translational modification (PTM) of proteins by tyrosine nitration (Arasimowicz-Jelonek and Floryszak-Wieczorek, 2011; Vandelle and Delledonne, 2011; Gaupels *et al*., 2011; Corpas *et al*., 2021; León, 2022). Importantly, the reaction between ONOO^–^ and G leads, among other things, to abundant formation of 8-nitroguanine (8-NG) (Jena and Mishra, 2007). At physiological pH, the process of 8-NG formation can reach significant levels within a few hundred milliseconds, suggesting that the process might be kinetically relevant *in vivo* (Sánchez *et al.*, 2022). The specific G modification via nitrating agents may target both nucleotides embedded in RNA and DNA and nucleotides that act as intracellular second messengers (Ihara *et al.*, 2011; Sawa *et al.*, 2013; Arasimowicz-Jelonek and Floryszak-Wieczorek, 2019; Petřivalský and Luhová, 2020) (Fig. 1). As demonstrated by Sánchez *et al.* (2022), 8-NG formed *in vitro* by the reaction between free G and ONOO^–^ remained stable for at least 4 h without spontaneous denitration. In turn, Masuda *et al*. (2002) revealed that during a 6 h incubation of both 8-NG and 8-oxo-Guo present in isolated RNA in phosphate-buffered saline (PBS) at 37 °C, only 5% of the modified nucleoside was lost. The pool of 8-NG in RNA was found to be much more stable than 8-nitro-2′-deoxyguanosine in DNA, which rapidly depurinates to release 8-NG (Masuda *et al*., 2002). At the cellular level, the stability of the nitro group could depend on the physicochemical environment surrounding the purine (Sánchez *et al.*, 2022).

During the past years 8-NG has been identified in diverse living systems including animals, plants, and microorganisms. Thus it has been recognized as a specific marker of nucleotide nitration in the cellular environment (Ihara *et al.*, 2011; Izbiańska *et al.*, 2018; Gajewska *et al.*, 2020). The first studies revealed that 8-NG is generated in significant amounts in isolated calf thymus DNA as a result of the action of various nitrating systems, and the G nitration may be of less quantitative importance than other forms of DNA damage (Tuo *et al*., 2000). Subsequent studies on 8-NG formation in nucleic acids were focused on the development of pathological conditions in animal models. Thus, increased 8-NG levels in RNA/DNA were noted in hamsters (*Cricetus cricetus* L.) in response to the infection with liver fluke (*Opisthorchis viverrini*) (Pinloar *et al.*, 2003), mice (*Mus musculus* L.) infected with the pneumotropic virus (Akaike *et al*., 2003), human gastric mucosa infected with *Helicobacter pylori* (Ma *et al.*, 2004), and in mice in response to arsenic exposure (Piao *et al.*, 2011). Currently, there are over a dozen reports on the nitration of nucleic acids in animal models (Murata *et al.*, 2012). On the other hand, information on the presence of 8-NG in other organisms is scarce.

To our knowledge, there is only one report on nitrated nucleic acids in microorganisms (Table 1). That study showed the accumulation of NO and ONOO^–^ in response to cadmium (Cd) in the oomycete plant pathogen, *Phytophthora infestans* (Mont.) de Bary, which was accompanied by a significant increase in 8-NG in RNA and DNA (Gajewska *et al.*, 2020). The highest 8-NG levels were noted at sublethal concentrations of Cd, indicating that these DNA/RNA lesions can contribute to the metal toxicity. As the 8-NG content in the RNA pool in *P. infestans* showed no statistically significant changes during moderate heavy metal stress, the authors proposed that the RNA nitrative modification might function as a swift adjustment of *P. infestans* metabolism under unfavourable conditions, rather than mere damage of RNA (Gajewska *et al.*, 2020). Interestingly, the 8-NG level in both healthy and stress-treated cells was similar in the RNA and DNA pools, indicating no differences in their susceptibility to the RNS-mediated nitration phenomenon.

**Table 1.** Reports on 8-nitroguanosine (8-NG) formation in plants and microorganisms

Species	Effects
**Plants**	
Potato plants (*Solanum tuberosum* L.)	↑ 8-NG in total RNA and poly(A)RNA in response to inoculation with *Phytophthora infestans* (Izbiańska *et al.*, 2018)
Apple embryos (*Malus domestica* Borkh)	↑ 8-NG in RNA in response to NO donors (Andryka-Dudek *et al.*, 2019)
Apple embryos (*Malus domestica* Borkh)	↓8-NG in RNA in response to NO fumigation and accelerated ageing (Ciacka *et al.*, 2021)
Longan (*Dimocarpus* longan cv. Daw)	↑ 8-NG during the process of pericarp browning in the post-harvested fruits (Intarasit *et al.*, 2022)
Maize (*Zea mays* L.)	↑ 8-NG in mRNA in response to aphids, more pronounced in relatively resistant Waza cultivar than in more susceptible Zlota Karlowa cultivar (Sytykiewicz *et al.*, 2024)
**Microorganisms**	
*Phytophthora* *infestans* (Mont.) de Bary	↑ 8-NG in RNA and DNA in response to cadmium (Cd) (Gajewska *et al.*, 2020)

↑, increase in the level; ↓, decrease in the level.

The first report on 8-NG in plants was related to pathophysiological conditions, as the marker was detected in potato (*Solanum tuberosum* L.) leaves challenge-inoculated with *P. infestans*, a causative agent of late blight disease (Izbiańska *et al.*, 2018). As shown in Table 1, 8-NG was also found in axes of embryos isolated from apple (*Malus domestica* Borkh) seeds, longan (*Dimocarpus longan* Lour) pericarp, and maize (*Zea mays* L.) seedlings (Andryka-Dudek *et al.*, 2019; Ciacka *et al.*, 2021; Intarasit *et al.*, 2022; Sytykiewicz *et al.*, 2024). In potato plants, inoculation with *P. infestans* accelerated 8-NG formation starting from the first hours post-inoculation (Izbiańska *et al.*, 2018). The level of 8-NG was detected in cultivars both resistant and susceptible to *P. infestans*, wherein this modification was ~5-fold higher in poly(A)RNA than in total RNA. The response was significantly more pronounced in the case of the resistant cultivar, where it coincided with ONOO^–^ accumulation and occurrence of the first symptoms of programmed cell death (PCD) during the hypersensitive response (HR). Accumulation of nitrated poly(A)RNA may provide a functional stage of metabolism reprogramming during pathogen perception and host signalling in incompatible (immune) interactions rather than being merely a consequence of cell death. Although the factors regulating poly(A)RNA nitration are unknown, the process seems to be selective (Izbiańska *et al*., 2018). As mRNA oxidation could compromise its translational activity and fidelity (Tanaka and Chock, 2021), targeted mRNA nitration might also lead to the diminished expression of specified proteins and thus constitute a mechanism of post-transcriptional gene expression regulation (Rivera-Mancía *et al*., 2017). Regarding biotic stresses, 8-NG accumulation in mRNA was also evidenced in maize seedlings exposed to aphid infestation (Sytykiewicz *et al.*, 2024). Similarly, as observed in potato response to avirulent *P. infestans*, RNA nitration in maize was also more pronounced in the aphid-resistant cultivar than in the more susceptible one. The response was time dependent, with the highest accumulation observed after 48 h and declining in the later periods. In addition, the same study revealed that 8-NG levels in mRNA were elevated under drought and combined stresses.

Studies on apple embryos suggest that RNA nitration might also be engaged in physiological processes. It is well documented that exposure of apple embryos to NO donors induced their germination (Gniazdowska *et al.*, 2010). The NO treatment also resulted in changes in the expression of several germination-related genes and 8-NG accumulation (Andryka-Dudek *et al.*, 2019). However, a subsequent study revealed that NO fumigation of embryos isolated from apple seeds subjected to accelerated ageing showed lowered nitro-oxidative modifications in the RNA pool. Thus, the authors recognized 8-NG as a marker of RNA damage and NO application as a remedy in oxidative remodelling after seed ageing (Ciacka *et al.*, 2021). Nitration of nucleic acids might also be associated with the process of post-harvest senescence. Study on longan revealed an association between RNS and pericarp browning in harvested fruits (Intarasit *et al.*, 2022). During the 7 d long storage, the RNS levels increased in longan fruits in a time-dependent manner, which was accompanied by pericarp browning and accumulation of the products of protein (3-nitrotyrosine, 3-NT), fatty acids (NO_2_-FA), and nucleic acid nitration (8-NG). Premature senescence of Arabidopsis (*Arabidopsis thaliana* L. Heynh) leaves was also accompanied by nitrative changes at RNA and DNA levels (Arasimowicz-Jelonek *et al*., 2022). The abundance of 8-NG in the RNA pool significantly increased on day 3 of dark-induced leaf senescence (DILS), while a significant rise of 8-NG DNA was noted on day 7 of DILS. The observed phenomenon of nucleic acid nitration was therefore defined as a part of developmental shifts during the life span of the plant, adjusting metabolism in a time-coordinated manner (Arasimowicz-Jelonek *et al*., 2022).

## Oxidative modifications of DNA

ROS are important signalling molecules engaged in various processes, including development and stress response (Mittler, 2017). Their level is tightly regulated by many elements comprising ROS-generating sites and ROS-scavenging antioxidants. It is estimated that in Arabidopsis >240 genes are involved in ROS metabolism (Oliveira *et al.*, 2019). Dysregulation of this system results in ROS overaccumulation and oxidative stress associated with oxidative damage of biomolecules. Nucleic acids are also susceptible to ROS action. Interaction between ROS and DNA leads to the formation of strand breaks, abasic (AP) sites, and oxidative modifications of nucleotides, of which 8-hydroxyguanosine (8-OHdG, -oxodG) is the most common. Formation of 8-OHdG can lead to mismatched pairing with adenosine and, as a consequence, an increased mutation rate (Sedelnikova *et al.*, 2010; Manova and Gruszka, 2015). In addition, it can cause a transient pause in RNA polymerase activity, leading to a slowing down of transcription (Tornalleti *et al*., 2004). Moreover, a study using minigene splicing reporters showed that 8-OHdG might lower splicing fidelity (Paredes *et al.*, 2017).

In the course of evolution, organisms have developed DNA repair systems. The DNA damage response (DDR) includes numerous elements, in which ATM (ataxia telangiectasia mutated) and ATR (ATM and Rad3-related) play crucial roles in the sensing of double-strand and single-strand DNA breaks, respectively. The activation of the signalling pathway results in stimulation of SOG1 (Suppressor Of Gamma 1) transcription factor, which in turn induces expression of genes engaged in DNA repair and cell cycle regulation. Although most DDR elements are conserved among eukaryotic organisms (including ATM and ATR), SOG1 is specific for plants. The DNA damage can result in cell cycle arrest, endoreplication, or activation of apoptotic pathways and cell death (Hu *et al.*, 2016; Szurman-Zubrzycka *et al.*, 2023). Interestingly, an association was found between the activation of DDR and the resistance of plants to pathogens (reviewed by Hu *et al.*, 2016). In Arabidopsis, SNI1 (suppressor of npr1-1, inducible 1) was recognized as a regulator of DDR signalling. Treatment with salicylic acid (SA) or mutation in SNI1 leads to the accumulation of DNA mutations and induction of defence genes, providing evidence for a link between SA and DDR signalling and acquisition of pathogen resistance (Yan *et al.*, 2013). DDR-mediated cell cycle arrest enables the repair of the damage before cell division. In the case of 8-OHdG formation, base excision repair (BER) is activated. In the first step of BER, altered nucleotides are removed by 8-oxoguanine glycosylase 1 (OGG1), forming an AP site. Thereafter, the AP site is removed by apurinic/apyrimidinic enodDNase (APE), and the gap is filled in by DNA polymerase (Sedelnikova *et al.*, 2010). Homologues of human OGG1 were found in plants (Dany and Tissier, 2001), yeast (Nash *et al.*, 1996), and in some bacterial species (Denver *et al.*, 2003). The excision of purines can also be carried out by MutM DNA glycosylase in bacteria (also referred to as formamidopyrimidine-DNA glycosylase, FPG) and by its homologue, MMH, in plants (Roldán-Arjona and Ariza, 2009). The metabolism of nitrative modifications of DNA is less studied, although the involvement of the BER system has been proposed, similarly to the case of oxidative modifications (Sánchez *et al.*, 2022). The presence of a flexible ring-opened structure in 5-guanidino-4-nitroimidazole (NIm), a product of peroxynitrite action, might be associated with thermodynamic destabilization of DNA duplexes (Jia *et al.*, 2006). The DNA duplexes containing NIm modifications were identified as effective substrates for the BER process (Shafirovich *et al.*, 2016). Intensified DNA oxidation has been observed in microorganisms and in plants under various unfavourable conditions. In bacteria, a concentration-dependent accumulation of 8-OHdG in response to H_2_O_2_ treatment was noted in *Escherichia coli* and *Salmonella typhimurium* (Park, 1990). Interestingly, ROS-driven changes in bacterial DNA can be associated with increased mutagenesis and acquisition of antibiotic resistance. Treatment with antioxidants resulted in decreased mutation rates in several bacterial species, such as *Bacillus subtilis*, *Staphylococcus aureus*, *Salmonella enterica*, and *Pseudomonas aeruginosa*. Further studies revealed that ROS-dependent accumulation of mutations and consequent antibiotic resistance depend on the nucleotide excision repair (NER) machinery (Carvajal-Garcia *et al.*, 2023). Similarly, in yeast, exposure to UVA induced the formation of 8-OHdG (Kozmin *et al.*, 2005). It is noteworthy that strains deficient in *OGG1* expression showed a higher mutation rate in response to UVA (Kozmin *et al.*, 2005) or H_2_O_2_ treatment (Melo *et al.*, 2004). In this context, it would be interesting to examine the potential involvement of 8-OHdG in the genomic changes and associated acquisition of virulence in plant bacterial and fungal pathogens.

In plants, higher numbers of DNA oxidation markers were noted in soybean (*Glycine max* L.) exposed to arsenic (Chandrakar and Keshavkant, 2019), in perennial ryegrass (*Lolium perenne* L.) treated with organic contaminants—phenol/polynuclear aromatic hydrocarbons (PAHs) (Malicka *et al*., 2021)—and in fenugreek (*Trigonella foenum graecum* L.) in reaction to lead (Pb), simulated acidic rain (SAR), or a combined Pb/SAR treatment (Xalxo and Keshavkant, 2018). In addition, a long-term experiment revealed that black locust (*Robinia pseudoacacia* L.), black alder (*Alnus glutinosa* L.), sycamore maple (*Acer pseudoplatanus* L.), and white willow (*Salix alba* L.) grown on metal-polluted soils amended with sulfo-calcic coal fly ashes (CFAs) exhibited elevated DNA oxidation levels when compared with unamended soil. In contrast addition of silico-aluminous CFA resulted in decreased 8-OHdG levels in *R. pseudoacacia*, *A. glutinosa*, and *S. alba* (Labidi *et al.*, 2017). The involvement of 8-OHdG in plant response to stress factors is also highlighted by the fact that Arabidopsis plants overexpressing *OGG1* show a higher germination rate in comparison with wild-type plants under methyl viologen (MV), mannitol, and salt treatment (Chen *et al.*, 2012). High expression of *OGG1* in developing and imbibing seeds indicates the important role of this enzyme in repairing DNA oxidative damage before and/or in the early stages of germination (Chen *et al.*, 2012; Ducatti *et al.*, 2022). An interesting characteristic of plants is the possession of OGG1 glycosylase, common in eukaryotes, and FPG glycosylase, which was perceived as typical for prokaryotes. In Arabidopsis, a double *OGG1*/*FPG* mutant showed a significant increase in nuclear and mitochondrial DNA damage, while in single mutants, the level of lesions was similar to that of the control. These results indicated that OGG1 and FPG are independently engaged in the repair mechanisms and can mutually compensate their activity (Córdoba-Cañero *et al.*, 2014). In addition, a study on barrel medic (*Medicago truncatula* Gaertn.) revealed that OGG1 and FPG are differentially regulated by copper (Cu) and polyethylene glycol (PEG) (Macovei *et al.*, 2011). Exposure to Cu induced expression both of genes in the roots and of *MtFPG* in the aerial parts. PEG treatment resulted in elevated expression of *MtFPG* in the roots and *MtOGG1* in the shoots. These results provide the evidence for the organ-specific activation of plant OGG1 and FPG.

## Oxidative modification of RNA

Similarly to 8-NG, the most frequent oxidative modification of RNA, 8-OHG, was initially studied in relation to the pathogenesis of human/animal disorders. Accumulation of this modification in RNA was observed in neurodegenerative diseases, cancer, diabetes, and ageing (Gan *et al.*, 2018; Li *et al.*, 2020).

8-OHG was also detected in microorganisms (Table 2). In *E. coli*, treatment with H_2_O_2_ led to an increase in 8-OHG levels in RNA but not in DNA, confirming the higher susceptibility of this nucleic acid type to oxidation (Liu *et al.*, 2012). The accumulation of 8-OHG was accompanied by RNA degradation and an increased rate of cell death. A study on an extremophilic bacterium *Deinococcus radiodurans*, exhibiting extraordinary resistance to oxidative stresses, provided insights into proteins binding to 8-OHG-enriched transcripts (Han *et al.*, 2022). Polynucleotide phosphorylase (PNPase), ATP-dependent helicase (RhlB), Rho, and ribosomal protein S1 (RpsA) showed selective binding to the oxidized RNAs, of which PNPase and RhlB exhibited the highest affinity. Decreased expression of *PNPase* and *RhlB* resulted in 8-OHG accumulation in RNA but not in DNA, suggesting the involvement of these proteins in the degradation of oxidized transcripts. What is noteworthy is that the mutated bacteria were less tolerant to oxidative stress. In turn, a study on yeast (*Saccharomyces cerevisiae* Meyen ex E.C. Hansen) revealed a connection between 8-OHG accumulation and ageing (Stirpe *et al.*, 2017). Yeast mutants deficient in the expression of the subunit of the decapping activator Lsm1-7 complex (*Kllsm4Δ1*) were characterized by higher 8-OHG levels in RNA, accelerated ageing, and an increased rate of mutations. This finding aligns with reports on higher RNA 8-OHG contents in the urine of elderly people (Gan *et al.*, 2018). Altogether, the reports on bacteria and yeast provide evidence for 8-OHG formation in RNA and point to its connection with ageing and cell death. Thus, RNA oxidation could also have significant impacts on microorganisms. However, to the best of our knowledge, this process has not yet been studied in plant beneficial, symbiotic, or pathogenic microorganisms. Nevertheless, the mechanism of resistance to nitro-oxidative stresses, to which the microorganisms are exposed when interacting with plants, could involve precise elimination of oxidized RNA molecules to efficiently adjust metabolism to the new host microenvironment.

**Table 2.** Reports on 8-hydroxyguanosine (8-OHG) detection in RNA of plants or microorganisms

Species	Effects
**PLANTS**	
Sunflower (*Helianthis annuus*)	↑ 8-OHG in transcripts of seeds subjected to dry after-ripening (Bazin *et al.*, 2011 )
Wheat (*Triticum aesticum* )	↑ 8-OHG in transcripts of seeds subjected to dry after-ripening (Gao *et al.*, 2013 ) ↑ 8-OHG in RNA in response to freezing (Jaikumar *et al.*, 2020)
Green alga (*Chlamydomonas reinhardtii*)	8-OHG-enriched RNA detected in chloroplasts (Zhan *et al.*, 2015)
Soybean (*Glycine max*)	↑ 8-OHG in total RNA and mRNA in response to Cd (Chmielowska-Bąk *et al.*, 2018 )
Arabidopsis (*Arabidospis thaliana*)	↑ 8-OHG in plants infected with the cyst nematode *Heterodera schachti* (Labudda *et al.*, 2018 )
Maize (*Zea mays L*.)	↑ 8-OHG in mRNA and total RNA, but not 8-OHdG in DNA in response to aphid infestation (Sytykiewicz *et al.*, 2019)
Arabidopsis (*Arabidospis thaliana*	↑ 8-OHG in ^1^O_2_ enriched environment (Koh *et al.*, 2021)
Soybean (*Glycine max*)	↑ 8-OHG in response to copper (Cu) and lead (Cd) (Chmielowksa-Bąk *et al.*, 2022)
**Microorganisms**	
*Escherichia coli*	↑ 8-OHG in response to H_2_O_2_ (Liu *et al.*, 2012)
*Deinococcus radiodurans*	↑ 8-OHG in response to H_2_O_2_ and ionizing radiation (Tsai *et al.*, 2015; Han *et al.*, 2022)
Yeast	↑ 8-OHG in *Kllsm4Δ1* mutants (Stirpe *et al.*, 2017)

↑, increase in the level.

In plants, RNA oxidation is implicated in physiological processes, specifically in the breakage of seed dormancy, as well as response to abiotic and biotic stress factors (listed in Table 2). In sunflowers (*Helianthus annuus* L.) and wheat (*Triticum aesticum* L.), an increase in 8-OHG was noted during alleviation of seed dormancy (Bazin *et al.*, 2011; Gao *et al.*, 2013). Those studies also revealed that RNA oxidation was selective and occurred in certain transcripts. In sunflower seeds, the highly oxidized transcripts were associated with cellular metabolism, stress response, and transport (Bazin *et al.*, 2011). In wheat seeds, the 8-OHG-enriched transcripts encoded proteins engaged in the biogenesis of ribosomes, oxidative phosphorylation, and metabolism of storage material (Gao *et al.*, 2013). The results led to two important conclusions. Firstly, the oxidation is transcript specific, but not species specific, as different transcripts were oxidized in sunflower and wheat seeds. Secondly, the oxidation of transcripts may be engaged in alleviating seed dormancy through regulation of the level of encoded proteins, for example engaged in the metabolism of the nutrient reservoir. This hypothesis is supported by the fact that oxidized transcripts are transcribed with lower efficiency, leading to a decreased accumulation of specific proteins (Bazin *et al.*, 2011; Gao *et al.*, 2013; Simms *et al.*, 2014; Koh *et al.*, 2021). In terms of seed biology, RNA oxidation was also studied during the process of ageing (Fleming *et al*., 2018). A comparison of soybean seeds differing in storage time revealed that ageing is associated with RNA non-enzymatic degradation. However, no correlation was found between the storage time and 8-OHG levels.

The accumulation of 8-OHG in RNA as a reaction to abiotic stress factors was first demonstrated in soybean seedlings treated with Cd (Chmielowska-Bąk *et al.*, 2018), followed by studies showing its induction in response to Cu and Pb (Chmielowska-Bąk *et al.*, 2022). Both studies documented that 8-OHG induction was an early reaction observed within 1–3 h of metal exposure. In contrast, the common oxidative stress markers were induced only after 24 h of treatment. The time-dependent changes in RNA oxidation were also evidenced in plant response to biotic stresses. In Arabidopsis plants infected with a cyst nematode, *Heterodera schachti* (Schmidt), the level of 8-OHG was the highest after 3 d of infection; it remained elevated after 5 d, and thereafter declined to the control levels. In contrast, lipid peroxidation was most intense at the later stages of infection (Labudda *et al.*, 2018). Another study compared the response of susceptible and relatively resistant (Zlota Karlova and Waza, respectively) cultivars of maize to bird-cherry-oat aphids (Sytykiewicz *et al.*, 2019). An increased RNA oxidation was noted after 24 h of infection in both cultivars, but it was significantly more pronounced in the case of the susceptible cv. Zlota Karlova. Interestingly, no changes were noted in the level of DNA oxidation, suggesting that RNA is more sensitive to the process. In terms of RNA types, poly(A)RNA showed ~5-fold higher 8-OHG levels than total RNA. The high susceptibility of poly(A)RNA to oxidation was also evidenced in other studies (Bazin *et al.*, 2011; Chmielowska-Bąk *et al.*, 2018). The stress-dependent oxidation of transcripts is dependent on plant species. The levels of 8-OHG in RNA were elevated in response to freezing in wheat but not in wheatgrass [*Thinopyrum intermedium* (Host) Barkworth & D.R. Dewey] or barley (*Hordeum vulgare* L.) (Jaikumar *et al.*, 2020). In addition, the level of 8-OHG in wheatgrass was generally higher in young than in older plants.

Various ROS types and sources could be responsible for RNA oxidation. In Arabidopsis, stimulation of ^1^O_2_ production by application of rose Bengal resulted in 8-OHG accumulation in total RNA and mRNA (Koh *et al.*, 2021). In turn, in soybean, Cd-dependent RNA oxidation declined slightly after the application of a mitochondria-specific antioxidant, MitoTEMPO (Fayazipour *et al.*, 2022). However, the MitoTEMPO treatment was also accompanied by decreased uptake of Cd. In a unicellular alga, *Chlamydomonas reinhardtii*, 8-OHG-enriched RNA was detected in the main ROS-generating organelle, namely the chloroplast. Interestingly, the knockout mutant with an attenuated expression of the Rubisco large subunit (RBCL) contained higher 8-OHG levels. The results indicate that RBCL is involved in the regulation of RNA oxidation (Zhan *et al.*, 2015).

## Biological effects of nitro-oxidative modifications of nucleic acids

At present it is difficult to state whether nitro-oxidative modifications are merely a symptom of nucleic acid damage or a sensing/regulative element, taking into account still limited information on their formation and effects. There have been several proposals for the damage-associated formation of 8-NG and 8-OHG (Fig. 3). Firstly, their accumulation is observed for various disorders in animal models (reviewed in Li *et al.*, 2020), ageing (Gan *et al.*, 2018; Intarasit *et al.*, 2022), and exposure to stresses in plants and microorganisms (Chmielowska-Bąk *et al.*, 2018, 2022; Izbiańska *et al.*, 2018; Labudda *et al.*, 2018; Gajewska *et al.*, 2020; Sytykiewicz *et al.*, 2024). Secondly, a higher accumulation of 8-OHG was noted in an aphid infection-susceptible maize cultivar rather than a resistant one, thus indicating its association with higher sensitivity to this biological stressor (Sytykiewicz *et al.*, 2019). In addition, RNA oxidation is associated with alterations in translation leading to translational errors, formation of truncated protein and aggregates, and ribosome stalling, negatively affecting the general cellular homeostasis (Shan *et al*., 2003; Tanaka *et al.*, 2007; Simms *et al.*, 2014; Calabretta *et al.*, 2015; Dai *et al.*, 2018).

**Fig. 2.**
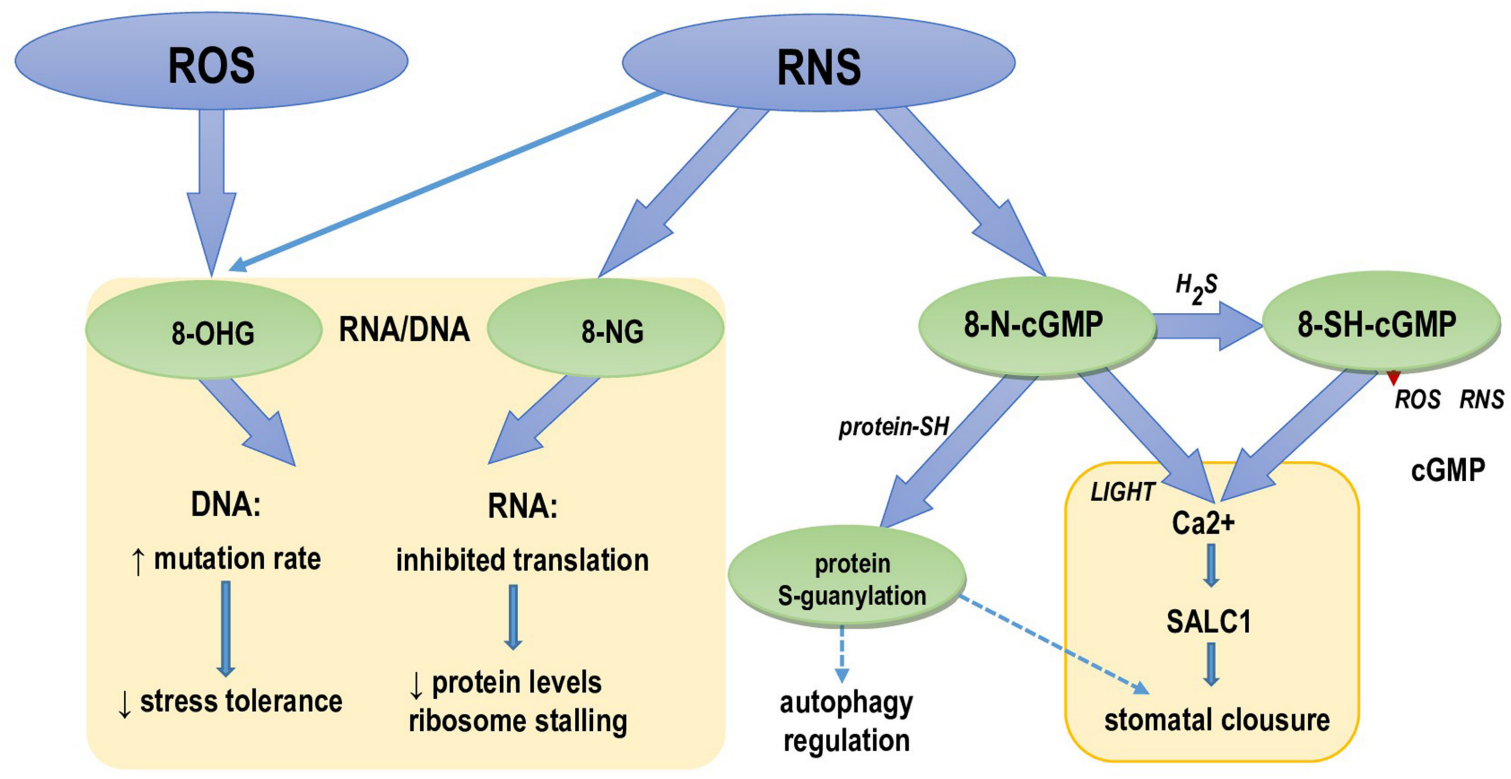
Potential effects of ROS/RNS-mediated modification of guanine nucleotides in plants. Formation of 8-NG and 8-OHG can lead to an increased rate of DNA mutations and hampered RNA translation. Nitro-cGMP and/or 8-SH-cGMP may induce cytoplasmic Ca^2+^ elevation and activate SLOW ANION CHANNEL1, leading to stomatal closure in light conditions. Nitro-cGMP via *S*-guanylation might also function as a protein PTM engaged in autophagy regulation.

**Fig. 3.**
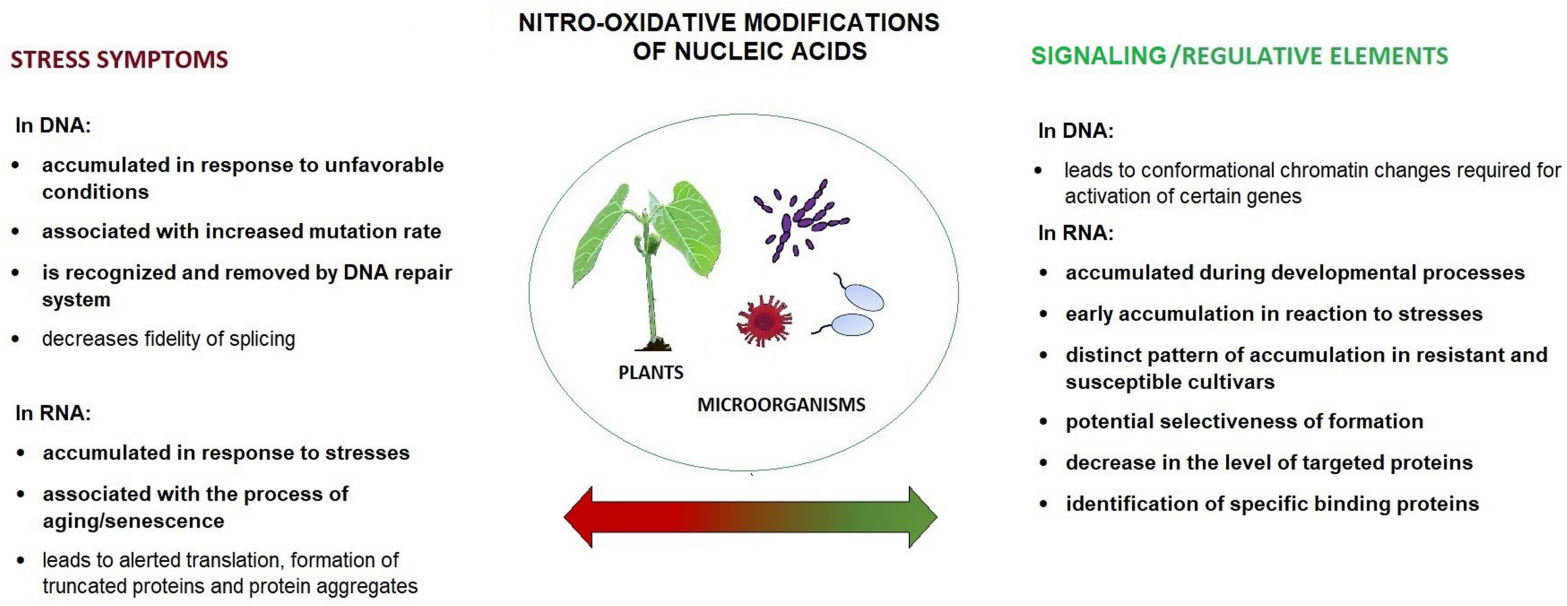
The proposed mechanisms for the formation of nitro-oxidative modifications of nucleic acids as a result of damage or signalling/regulative elements. Processes marked in bold have been observed in microorganisms and plants.

On the other hand, an increase in the level of nitrated and oxidized transcripts was also described during a physiological process, such as interruption of seed dormancy (Bazin *et al.*, 2011; Gao *et al.*, 2013; Andryka-Dudek *et al.*, 2019). In addition, the time-dependent changes in the 8-NG and 8-OHG levels observed in plants in response to biotic/abiotic stress factors imply some mechanisms of their regulation (Chmielowska-Bąk *et al.*, 2018, 2022; Izbiańska *et al.*, 2018; Labudda *et al.*, 2018; Sytykiewicz *et al.*, 2019, 2024). Indeed, in bacteria, PNPase and RhlB are indicated as proteins controlling the degradation of oxidized transcripts (Han *et al.*, 2022). In turn, in animal/human models, Y-box-binding proteins 1 (YB-1) (Hayakawa *et al.*, 2002), humans heterogeneous nuclear ribonucleoproteins known also AU-rich element/poly(U)-binding/degradation factor 1 (HNRNP/AUF1) (Hayakawa *et al.*, 2010), and poly(C)-binding protein 1 (PCBP1) (Ishii *et al*., 2018) were shown to bind to oxidized transcripts. AUF1 is likely to be engaged in the susceptibility of oxidized RNA to degradation. In turn, PCBP1 recognizes heavily oxidized transcripts and initiates the programmed cell death pathway. Thus, the heavily 8-OHG-enriched transcripts can carry information on severe oxidative stress and activate the radical defence mechanisms. Another possible 8-OHG-dependent signalling pathway has been described in Arabidopsis (Koh *et al.*, 2021). In that study, a correlation was found between high levels of 8-OHG in transcripts and attenuated expression of the labile gene repressors. Release from their repression will lead to the higher expression of dependent genes. Importantly, several reports have indicated that RNA oxidation is a selective process occurring in specific transcripts (Shan *et al.*, 2003; Chang *et al.*, 2008; Bazin *et al.*, 2011; Gao *et al.*, 2013). In post-mortem brain tissues of patients suffering from Alzheimer’s disease, the oxidation did not occur in the most abundant transcripts, such as those encoding actin, neurofilament light chain protein, or glutamate transporter. In turn, several 8-OHG-enriched transcripts encoded proteins associated with detoxification processes or cellular signalling, such as p21ras, superoxide dismutase (SOD), transferrin, mitogen-activated protein kinase (MAPK), or calpain (Shan *et al.*, 2003). The higher susceptibility of specific transcripts to oxidation was also confirmed using microarrays, for example in mice (Chang *et al.*, 2008), and sunflower (Bazin *et al.*, 2011) and wheat seeds (Gao *et al.*, 2013). Illumina sequencing of oxidized transcripts from human bronchial epithelial BEAS-2B cells exposed to formaldehyde revealed that oxidation occurred in RNA belonging to specific functional categories, in particular chromatin regulation and DNA repair (Gonzales-Rivera *et al.*, 2020). These results further support the hypothesis on the selectiveness of oxidation.

The hampered translation of 8-OHG-enriched transcripts and the resulting decrease in encoded protein levels might constitute a mechanism of post-transcriptional gene expression regulation. The sensing/gene regulatory role of 8-OHG-bearing transcripts is further supported by their early accumulation in plants in response to metals, preceding the accumulation of oxidative stress markers (Chmielowska-Bąk *et al.*, 2018, 2022). It might also suggest that as RNS and ROS have been produced by organisms for millions of years, the most frequent by-products of their action, such as oxidized transcripts, have acquired some biological roles.

The exact biological effects of nitro-oxidative modifications probably depend on RNS/ROS concentrations and the type of nucleic acid. It is logical to assume that DNA, as a store of genetic material, should be more stable in varying internal and external conditions. Thus, RNS/ROS-dependent, non-enzymatically formed modifications, which are not subjected to tight control, are generally perceived as symptoms of damage (Sedelnikova *et al.*, 2010; Lonkar and Dedon, 2011; Manova and Gruszka, 2015). However, it was proposed that even oxidative modifications of DNA could carry out some gene regulatory functions (Fleming and Burrows, 2017). It was noted that 8-OHdG levels were higher in euchromatin than in heterochromatin. Moreover, DNA oxidation in the promoter region of the gene encoding vascular endothelial growth factor A (*VEGF*) induced its expression. A more detailed study revealed that 8-OHdG formation in the *VEGF* promoter leads to its removal by OGG1 and subsequent formation of the AP site. The AP site is further removed by the APE enzyme. The described sequence of events leads to conformational changes in chromatin, activating the *VEGF* gene (Fleming *et al.*, 2017). In mice MLE-12 cells (immortalized type 2 mouse lung epithelial cell line), 8-OHdG and OGG1 were shown to facilitate binding with nuclear factor kappa B (NF-κB) transcription factor. OGG1 was required for NF-κB-dependent activation of downstream genes (Pan *et al.*, 2017). In contrast, 8-OhG formation of the NEIL (Nei-like) promoter sequence stabilized the G-quadruplex (G4) structure, leading to hampered replication (Omaga *et al.*, 2018). Thus, the formation of 8-OHdG in DNA and the recruitment of proteins engaged in its processing might lead to conformational changes of chromatin, which in turn affect its accessibility for the transcription/replication machinery (Hahm *et al.*, 2022). In addition, an inter-relationship was found between 8-OHdG formation and epigenetic markers. A study on the plant pathogen *Botrytis cinerea* showed an association between histone demethylase activity and ROS production (Hou *et al*., 2020). It is proposed that the demethylation-dependent ROS production, accompanied by localized 8-OHdG formation and OGG1 recruitment, is engaged in oestrogen and androgen signalling in human cells (Perillo *et al.*, 2008; Yang *et al.*, 2015). On the other hand, the presence of 8-OHdG in DNA affects its methylation. *In vitro* assays showed that the incorporation of methyl groups is hampered in oxidized oligonucleotides (Weitzman *et al.*, 1994). In addition, this modification decreased the binding affinity of methyl-CpG binding proteins (MBPs), which regulate chromatin condensation (Valinluck *et al.*, 2004).

In contrast to DNA, transcripts are more transient, which makes them suitable candidates for signal-responsive and/or regulatory elements. Studies on m6A and 5mC showed that modified nucleotides affect the stability (Wei *et al.*, 2018), translocation (Yang *et al.*, 2019), and translation efficiency (Wang *et al.*, 2023) of transcripts participating in the regulation of developmental processes (Vespa *et al*., 2004; Bodi *et al.*, 2012; Wei *et al.*, 2018) and stress response (Tang *et al.*, 2020; Wang *et al*., 2023). Thus, nitro-oxidative modifications could also act as sensors of changes in RNS/ROS equilibrium, which in turn is modulated by various internal and external stimuli. This hypothesis is supported by the above-mentioned accumulation of 8-NG and 8-OHG during the physiological process of interrupting seed dormancy, time-dependent regulation of 8-NG and 8-OHG levels in response to stresses, and potential selectiveness of 8-OHG formation. Based on the pattern of the accumulation of nitro-oxidative modifications in transcripts, the prime areas of further research could focus on their possible involvement in plant stress response, seed germination, and seed priming. Seed priming is defined as application of pre-sowing agronomic techniques aiming at enhancement of seed vigour, frequently also associated with higher stress tolerance (Pagano *et al*., 2023). Several studies show that pre-treatment of seeds with H_2_O_2_ increases seed germination rate and seedling growth, and/or alleviates the effects of stress conditions (Savvides *et al.*, 2016; Dufková *et al.*, 2019; Habib *et al*., 2021; Tahjib-Ul-Arif *et al.*, 2024). In eggplant (aubergine; *Solanum melongena* L.), the enhanced germination of H_2_O_2_-pre-treated seeds was accompanied by proteomic changes (Dufková *et al*., 2019), which in turn could be regulated by oxidation of transcripts as described earlier in sunflower and wheat seeds (Bazin *et al*., 2011; Gao *et al*., 2013). Similarly, pre-treatment of seeds with the NO donor sodium nitroprusside (SNP) increased the growth parameters of wheat in optimal conditions and under salt stress (El-Shazoly *et al.*, 2024). In addition, SNP seed pre-treatment resulted, for example, in higher tolerance of germinating maize seedling against methylgloxal toxicity (Yiğit and Atici, 2022). Thus, assessment of the possible role of nitro-oxidative modifications of transcripts in boosting the vigour and stress tolerance of ROS/RNS-primed seeds could be an interesting area of study.

However, further research is needed to fully confirm the regulative roles of nitrated and oxidized transcripts. One of the major challenges would be to identify proteins engaged in the metabolism of nitro-oxidative ribonucleotides in plants and microorganisms, bringing us closer to the acquisition of 8-NG- or 8-OHG-deficient mutants. Moreover, the mechanisms responsible for RNA oxidation selectiveness are still unknown. The possible selectiveness of the nitration process has not been evidenced at all due to the lack of nitrotranscriptomic data. In addition, certain modifications can influence the formation of others, as observed, for example, in the case of 8-OHdG formation in DNA and cytosine methylation. Thus, interactions between various modifications in DNA and RNA are another still poorly examined area of epigenetics and epitranscriptomics. Furthermore, the studies are focused on the most frequently modified nucleotides, 8-OHG and 8-NG. However, there are multiple other nitro-oxidatively modified nucleotides (Barciszewski *et al.* 1999; Roldán-Arjona and Ariza, 2009), which could also impact the functioning of microorganisms and plants. Another intriguing aspect is the pattern of the formation of modified nucleotides in specific cellular compartments. Some modifications, such as m6A, 5mC, and pseudouridine in RNA (reviewed in Adamiec and Luciński, 2024) or 5-mC in DNA (Takio *et al*., 1994; Scheschonk *et al*., 2024), were detected in chloroplasts. In addition, studies on Arabidopsis mutants indicated that RNA methylation is important for efficient chloroplast functioning, in particular under stress conditions (reviewed in Adamiec and Luciński, 2024). Thus, chemical modifications of nucleic acids might not be limited to the nucleus and cytoplasm but may also play a role in other cellular compartments. Taking into account the spatial diversification of RNS/ROS sources, which, depending on the reactive species type, comprise the chloroplast, mitochondria, nucleus, cellular membranes, cell walls, and/or cytoplasm (Shapiguzov *et al*., 2012; Astier *et al*., 2018; Karpinska and Foyer, 2024), it is highly likely that the pattern of the nitration/oxidation of nucleic acids is site specific. The hypothesis on DNA oxidation in specific organelles, such as chloroplasts, is supported by the identification of chloroplast glycosylases mediating BER of damaged DNA (Gutman and Nyiogi, 2009). The possibility of interplay between the nitro-oxidative status of one cellular location and the level of DNA/RNA modifications in another compartment further contributes to the complexity of the picture. For instance, studies on Arabidopsis *apx1/cat2* mutants, with decreased expression of peroxisomal catalase and cytosolic ascorbate peroxidase, showed constitutive expression of genes associated with DDR and diminished levels of DNA oxidative alterations (Vanderauwera *et al*., 2011). The RNS/ROS network is even more complicated by numerous elements engaged in its scavenging, such as antioxidant enzymes and non-enzymatic antioxidant compounds. Several reports show no or even negative association between the level/activity of antioxidant enzymes and plant stress tolerance, supporting the claim that certain levels of ROS are important for stress response (Xu *et al.*, 2024). Thus, manipulating RNS/ROS sources and/or scavengers under unfavourable conditions could provide information on a possible link between RNS/ROS-localized accumulation, consequent formation of nitro-oxidative modifications of nucleotides, and stimulation/attenuation of defence mechanisms. In conclusion, despite the significant progress made in nitro-oxidative modifications, there are still many open questions to be answered (summarized in Box 1).

**Box 1.** Highlighted open questions in the area of nitro-oxidative modifications in plants and microorganisms.

Open questions	
**What is the role of specific modifications in plant developmental processes?**	Individual reports show 8-NG and 8-OHG accumulation during alleviation of seed dormancy or seed germination (Bazin *et al*., 2011; Gao *et al*., 2013; Andryka-Dudek *et al.*, 2019; Ciacka *et al.*, 2021). In addition, 8-NG levels increased by the post-harvest senescence of fruits (Intarasit *et al.*, 2022). It would be interesting to monitor the levels of these modifications during plant development and explore their possible regulative roles.
**Is the formation of 8-OHG and 8-NG a universal response to the stress factors?** **What are their possible roles in the acquisition of stress tolerance?** **How can these modifications affect plant–pathogen interactions?** **Do these modifications benefit the host plant or the pathogen?**	Exposure to drought, metals, pathogens, and herbivores induces formation of nitro-oxidative modifications of RNA in plants (Chmielowska-Bąk *et al.*, 2018, 2022; Izbiańska *et al.*, 2018; Labudda *et al.*, 2018; Sytykiewicz *et al.* 2019, 2024; Gajewska *et al.*, 2020). However, so far it is not clear if these modifications are a symptom of stress-dependent damage or a sensing/regulative element.
**Could 8-OHG and/or 8-NG act as signals in communication between plants and beneficial microbes?**	NO/ROS function as a signals triggering several essential mechanisms occurring in plant–microbe association (Boscari *et al.*, 2013; Hichri *et al.*, 2015). However, the possibility of the involvement of nitro-oxidative modifications in nucleotides during the beneficial relationships remains unrecognized.
**Is DNA oxidation and/or nitration solely a symptom of damage?**	Initially, DNA oxidation or nitration was perceived as damage to the genetic material. However, some studies point out the possible involvement of 8-OHdG in the activation of specific genes (Fleming *et al.*, 2017; Hahm *et al.*, 2022). The possibility of the involvement of DNA nitro-oxidative modifications in gene regulation (e.g. on an epigenetic level) would need further elucidation.
**Is 8-OHG and 8-NG formation in the transcripts selective?**	The selectiveness of 8-OHG formation was indicated in several studies on plant and animal models (Shan *et al.*, 2003; Chang *et al.*, 2008; Bazin *et al.*, 2011; Gao *et al.*, 2013). However, the reports are still limited. Moreover, there is no confirmation of selectiveness during exposure to stresses when the nitro-oxidative status of an organism can be disturbed. In addition, there is no evidence for selective formation of 8-NG.
**Are the 8-OHG and 8-NG levels regulated by specific proteins?**	Specific proteins exhibiting high binding affinity for 8-OHG-enriched RNA were identified in microorganisms and humans, wherein some are engaged in the degradation of oxidized transcripts or activation of cell programmed death (Hayakawa *et al.*, 2002, 2010; Ishii *et al*., 2018). However, no analogues of such 8-NG/8-OHG-binding proteins were identified in plants.
**Is 8-OHG and 8-NG formation tissue and organelle specific?** **Can these modifications carry out specific functions depending on their localization?**	The RNS/ROS network comprises numerous inter-related elements localized in distinct cellular compartments (Shapiguzov *et al.*, 2012; Astier *et al.*, 2018; Karpinska and Foyer, 2024). Therefore, it is highly likely that the pattern of nitro-oxidative modifications is site specific. It would be particularly interesting to examine their role in organelles showing dynamic RNS/ROS metabolism and their own set of nucleic acids—chloroplast and mitochondria.
**What is the impact of the less studied modifications on the functioning of organisms?**	The majority of the studies are focused on the most frequent nitro-oxidative modifications, 8-NG, 8-OHG, and 8-N-cGMP. Simultaneously, RNS/ROS action can lead to the formation of numerous other modified nucleotides. Exploration of their presence, metabolism, and role in microorganisms and plants could significantly broaden our knowledge of epitranscriptomic and epigenetic regulations.

## Biological effects of nitro-oxidative modifications of cyclic nucleotides

Signalling via the ⋅NO/cGMP pathway is engaged in several crucial physiological processes within all the kingdoms of life. Notably, the nitro-oxidative environment can create a membrane-permeable 8-nitroguanosine-cGMP (8-N-cGMP); however, the formation of 8-N-cGMP results from the nitration of abundant GTP, which then is metabolized into 8-N-cGMP by soluble guanylate cyclase (Sawa *et al.*, 2007; Fujii *et al.*, 2010; Kunieda *et al.*, 2015). The degradation route of 8-N-cGMP involves sulfhydration into 8-mercapto-cGMP (8-SH-cGMP) through a reaction with endogenous persulfides (Fig. 2). The sulfhydryl group of 8-SH-cGMP is eliminated, giving rise to the formation of cGMP, which promotes hydrolysis by phosphodiesterases into GMP. As 8-N-cGMP was detected at a relatively high level (≥40 μM) compared with cGMP (4.6 μM) in rat glioma C6 cells, the nitrated cGMP derivative was considered as the major intracellular cyclic nucleotide (Fujii *et al.*, 2010). In addition, 8-N-cGMP was proven to possess the strongest redox-active and electrophilic properties among the nitrated guanine derivatives (Ihara *et al*., 2011). The unique features underlie the ability of 8-N-cGMP to react with highly nucleophilic sulfhydryl groups of cysteine residues of redox sensor proteins to form a protein–S-cGMP adduct via S-guanylation. There are several identified target proteins of *S*-guanylation, including Kelch-like ECH-associated protein 1, H-Ras, mitochondrial heat shock protein 60, cGMP-dependent protein kinase, synaptosomal-associated protein 25, and tau protein (reviewed by Kasamatsu *et al*., 2016). Moreover, *S*-guanylation proteomics allow the identification *S*-guanylation protein targets in *E. coli*, including chaperones, ribosomal proteins, and enzymes which are associated with protein synthesis, redox regulation, and metabolism (Tsutsuki *et al*., 2016). Thus, signalling via 8-N-cGMP by *S*-guanylation of target proteins can constitute a specific imprint of an adaptive response to unfavourable conditions (Sawa *et al.*, 2013).

It is worth noting that bacterial infection may accelerate the intracellular formation of 8-N-cGMP in host cells. The accumulated 8-N-cGMP may contribute to antibacterial autophagy, as documented in the pathosystem involving a group A streptococcus, a leading human bacterial pathogen with diverse clinical manifestations, and murine macrophages (Ito *et al.*, 2013). The nitrated cGMP regulated autophagy via protein *S*-guanylation, as a significant correlation between the PTM and Lys63-linked ubiquitination level at the surface of the group A streptococcus was observed. Thus, the *S*-guanylation, abundant around invading bacterial cells, functions as a ‘tag’ for subsequent organism clearance via ubiquitin modifications (Arimoto and Takahashi, 2017). Importantly, bacteria can interfere with autophagy-mediated pathogen clearance via the production and release of reactive persulfides, which inhibit autophagy signalling by degrading 8-N-cGMP in host cells (Khan *et al.*, 2018).

In plants, 8-N-cGMP has been characterized as a novel second messenger involved in abscisic acid- and NO-induced stomatal closure (Joudoi *et al.*, 2013). Joudoi *et al.* (2013) documented that the overproduction of NO and ROS enhanced 8-N-cGMP formation in Arabidopsis guard cells, resulting in stomatal closure. Importantly, the effects mediated by cGMP and 8-N-cGMP are contrasting in character, as cGMP causes stomata to open in the dark, whereas 8-N-cGMP causes stomata to close in the light. Thus, both NO/nitrated cGMP and the classical NO/cGMP signalling cascades operate in guard cells. Using an inhibitor and *abi1-1* mutants, those authors suggested that calcium, cyclic ADP-ribose, and SLOW ANION CHANNEL1 act downstream of 8-N-cGMP (Honda *et al.*, 2015).

In contrast to animal systems, where sulfhydration of 8-N-cGMP inhibits the *S*-guanylation of specific proteins and is thought to be required for terminating electrophile-mediated signalling (Nishida *et al.*, 2012), in plant guard cell signalling, 8-mercapto-cGMP triggers the same signalling cascade (i.e. the cADPR/Ca^2+^/SLAC signalling pathway) as 8-nitro-cGMP and shows higher activity than nitrated cGMP. Thus, the reaction of 8-N-cGMP sulfhydration was documented as a promoting step for plant guard cell signalling (Joudoi *et al.*, 2013).

## Conclusions and future perspectives

Future research on nitro-oxidative modification of nucleic acids should be accompanied by methodological developments, as current studies are subjected to certain constraints. The standard methods for quantification of modified nucleotides comprise chromatographic techniques and antibody-based ELISA. Comparison of GC-MS, HPLC with electrochemical detection (HPLC-ECD), and HPLC with tandem MS (HPLC-MS/MS) for DNA 8-OHdG detection carried out by The European Standards Committee on Oxidative DNA Damage (ESCODD) across various laboratories revealed significant variation in the results (Collins *et al*., 2024). Despite analysis of the same material, the 8-OHdG content differed by even three magnitudes, possibly due to oxidation of the DNA during preparative steps. On the other hand, 8-OHdG quantification using ELISA tests resulted in 6-fold higher values when compared with HPLC (Yin *et al.*, 1995). This could result from higher sensitivity or cross-reactivity with other bases (Park *et al.*, 1992; Yin *et al.*, 1995). The cross-reactivity of the antibodies could also impact the sequencing of immunoprecipitated transcripts. This problem could be resolved by the application of direct RNA sequencing, for example through Oxford Nanopore (Wheeler *et al*., 2024). The methods are promising, as a report on the successful application of the training model for nanopore 8-OHdG detection in DNA has been recently published (Pagès-Gallego *et al.*, 2024, Preprint). Another impediment is the non-enzymatic formation of nitro-oxidative modifications of nucleotides. The RNS/ROS-mediated formation of these modifications shapes the intracellular redox state, mediating redox signalling. In consequence, ROS/RNS metabolism is regulated by a vast amount of elements/genes, complicating the elaboration of 8-NG/8-OHG-deficient mutants. Thus, there is a need for protocols facilitating the identification of putative 8-NG- and 8-OHG-binding proteins, which could be used to regulate their levels.

Nevertheless, increasing evidence documents the formation of nitro-oxidative modifications in RNA and DNA in plants and microorganisms of heterogeneous systematic positions. Moreover, the formation of the nitrated cGMP derivative, a novel second messenger in plants, indicates that biological effects of the nucleotide modifications are not only a damage symptom but possibly function as a sensing/regulative element of the complex redox signalling network. Further work is needed to elucidate the exact impact of modified nucleotides on the metabolism of nucleic acids and the cGMP pathway, along with their possible involvement in plant/microorganism functioning under physiological and stress conditions. An interesting aspect is also connected with the potential involvement of RNS/ROS-dependent modifications in various plant–microorganism interactions, as the available reports indicate their differentiated formation during effector-triggered immunity and disease development.

## Abbreviations

BER: base excision repair
DDR: DNA damage response
m6A: *N* ^6^-methyladenosine
5mC: 5-methylcytosine
8-NG: 8-nitroguanosine
8-OHG: 8-hydroxyguanosine
ROS: reactive oxygen species
RNS: reactive nitrogen species
